# Intrathoracic scapular dislocation following lung cancer resection

**DOI:** 10.1093/jscr/rjy178

**Published:** 2018-07-21

**Authors:** Masaki Tomita, Ayaka Iwasaki, Takanori Ayabe, Ryo Maeda, Kunihide Nakamura

**Affiliations:** 1 Department of Thoracic and Breast Surgery, Faculty of Medicine, University of Miyazaki, Kihara 5200, Kiyotake, Miyazaki, Japan; 2 Department of Cardiovascular Surgery, Faculty of Medicine, University of Miyazaki, Kihara 5200, Kiyotake, Miyazaki, Japan

## Abstract

A 64-year-old man underwent right upper lobectomy combined resection with third-fifth rib for lung cancer and reconstruction of chest wall using Dual Mesh. Six days after surgery, he experienced acute severe pain in the right shoulder. The purulent drainage through the drainage tube was also found. Chest CT showed that the inferior angle of the scapula protruded into the right intrathoracic cavity. We performed a removal of Mesh. Although we did not want to use synthetic materials because of infection, we performed titanium plate fifth rib fixation to avoid the recurrent dislocation of the scapula. After the redo surgery, continuous lavages with physiologic saline of the thoracic cavity was also performed. Patient is now doing well without recurrences of cancer, infection and scapular dislocation, 14 months after the redo surgery.

## INTRODUCTION

An intrathoracic dislocation of the scapula after lung cancer resection is a rare complication. Only some limited cases have previously been reported of intrathoracic dislocation of the scapula, in which the scapula becomes caught inside the ribcage, following thoracotomy [1–6]. We herein present a case who required a redo surgery for an intrathoracic scapular dislocation after lung cancer resection and combined chest wall resection. Furthermore, because this case complicated a local infection, the consideration of treatment plan was confusing.

## CASE REPORT

A 64-year-old man who underwent a right upper lobectomy in our institute, involving an extended resection of the posterior chest wall to treat a stage IIIA lung cancer. The dorsal portion of the third to fifth rib was additionally cut during the procedure, and the defect of chest wall was covered using Gore-Tex^®^ Dual Mesh (Japan Gore-Tex Inc., Tokyo, Japan), which is a pure and unique expanded polytetrafluoroethylene (ePTFE) prosthesis, consists of two functionally distinct surfaces [7].

**Figure 1:**
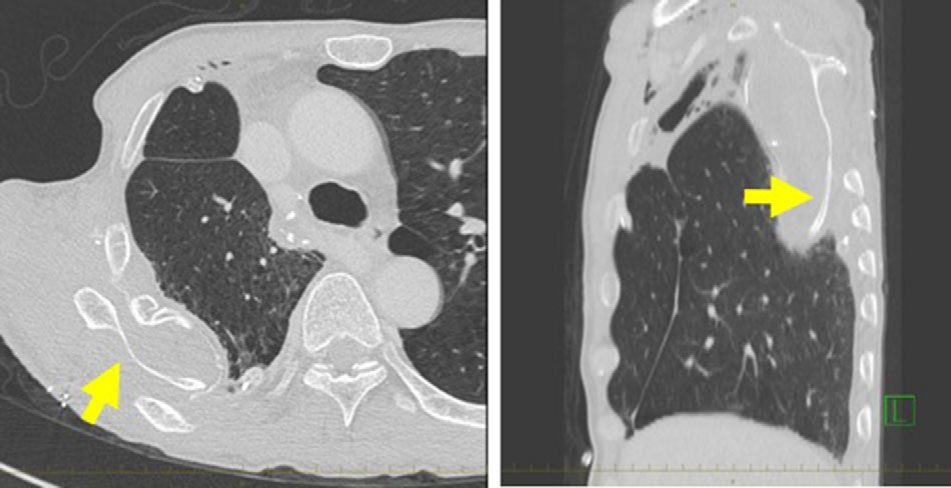
(**A**) Computed tomography (CT) demonstrating a dislocation of the right scapula in the right thoracic cavity. (**B**) Sagittal reconstruction of the CT also showed the dislocation of the right scapula.

**Figure 2:**
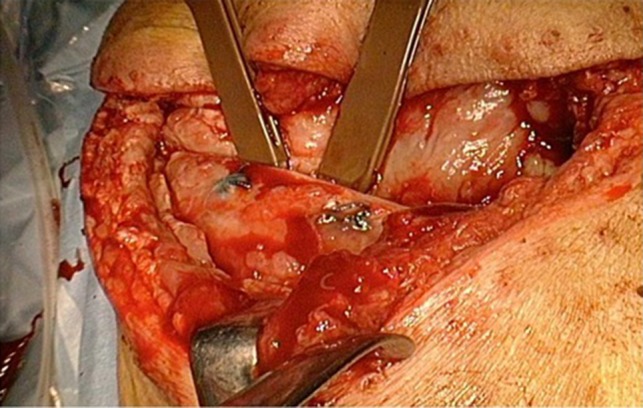
Intraoperative finding. The dislocation of the right scapula with the inferior angle of the scapula protruding into the right intrathoracic cavity though his thoracotomy defect.

**Figure 3:**
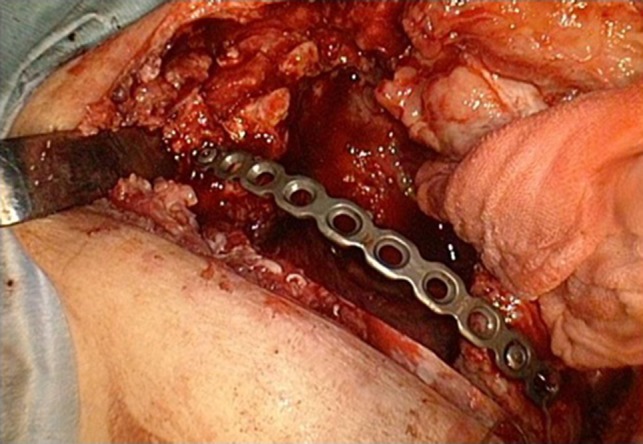
The fixation of fifth rib using titanium plate.

Postoperative acute course was uneventful. However, at postoperative Day 6, he experienced acute severe pain in the right shoulder. The slightly purulent drainage through drainage tube positioned on the mesh was also found. Pus culture from the drain discharge isolated Corynebacterium atrium. Computed tomography (CT) revealed that when the symptoms appeared, abnormal position of the right scapula with the inferior angle of the scapula was caught inside the top of the sixth rib (Fig. 1). The conservative treatment failed to improve the scapular dislocation. We performed a redo surgery. We found that all suture threads which attached Mesh to sixth rib were cut, and the dislocation of the right scapula with the inferior angle of the scapula protruding into the right intrathoracic cavity though his thoracotomy defect (Fig. 2). We also found a local infectious change at the head side of the Mesh (not the site of scapular dislocation), and found no macroscopic intrathoracic infectious changes. A removal of mesh was carried out. After improving the scapular dislocation and removal of mash, washing with 10 L of physiologic saline was carried out. We did not want to use synthetic materials because of infection. In this case, however, the infection was localized and titanium plate is reported to be resistance to infection [8], we performed titanium plate fifth rib fixation to avoid the recurrent dislocation of the scapula (Fig. 3). After the redo surgery, continuous lavages with physiologic saline of the thoracic cavity and antibiotic therapies were also performed for 21 days. The repeated culture of the drainage showed no bacterial isolation. After removal of drainage tube, there was no recurrence of infection and scapular dislocation. He had full range of motion of his right shoulder without pain. Patient is now doing well with free from recurrences of cancer, infection and scapular dislocation, 16 months after the redo surgery.

## DISCUSSION

An intrathoracic dislocation of the scapula after lung cancer resection is a rare complication. There are only some previous reports of an intrathoracic dislocation of the scapula following thoracotomy and/or chest wall resection [1–6]. These reports showed the intrathoracic dislocation of the scapula though the intercostal space of thoracotomy [1–4] or the chest wall defect after rib resection [5, 6]. In the present case, although we performed chest wall reconstruction, all suture threads which attached Mesh to sixth rib were cut and the scapular dislocation occurred. We believe that the major reason for this scapular dislocation was not due to use of Dual Mesh. We reflected that we should have a little more tightly and strongly attached Mesh, considering the risk of the intrathoracic dislocation of the scapula. Although the present case also complicated local infection, we also believe that the infection is not the reason for the dislocation of the scapula because the site of infection and the scapular dislocation was different.

In case with infection, traditional surgical teaching advocates the removal of all the potential sources of infection (removal of all synthetic materials). Moreover, it is recommended not to use further synthetic materials. One of possible operative procedure for this condition is the removal of inferior angle of the scapula without chest wall reconstruction. Kimura and Sasanuma [6] carried out this procedure. However, they reported that the patient’s pain still occurred when the resection margin collided with the ribs postoperatively [6]. Although they considered another operation, the decision was made to implement palliative care because localized recurrence of the lung cancer was present and metastases had enlarged [6]. Another possible procedure is the scapulothoracic arthrodesis [9]. However, this procedure is a recognized treatment for impaired shoulder function in patients with facioscapulohumeral dystrophy [9]. Additionally, this procedure has been reported to have postoperative complications such as brachial plexus palsy, restriction of range of motion and persistent pain [10].

Generally, it is recommended to reconstruct the chest wall not to use synthetic materials in case with infection. However, the titanium plate is reported to be resistance to infection [8, 10]. Berthet *et al*. [8] have shown that removal of the titanium bars does not seem to be mandatory in cases of infection after chest wall reconstruction. They concluded that the generally accepted notion that ‘deep infection of thoracic reconstruction requires hardware removal’ should perhaps be re-examined when titanium has been used [8]. Since we afraid the recurrence of infection following titanium plate rib fixation, we performed the continuous lavages and antibiotic therapies for ~3 weeks. We should examine an appropriate treatment period not to let infection recur.

## CONCLUSION

We presented a rare case who required a redo surgery for an intrathoracic scapular dislocation after lung cancer resection and combined chest wall resection. Surgeons should consider the risk of the intrathoracic dislocation of the scapula when attach the Mesh for the chest wall defect.
